# Differences in Shedding of the Interleukin-11 Receptor by the Proteases ADAM9, ADAM10, ADAM17, Meprin α, Meprin β and MT1-MMP

**DOI:** 10.3390/ijms20153677

**Published:** 2019-07-26

**Authors:** Martin Sammel, Florian Peters, Juliane Lokau, Franka Scharfenberg, Ludwig Werny, Stefan Linder, Christoph Garbers, Stefan Rose-John, Christoph Becker-Pauly

**Affiliations:** 1 Institute of Biochemistry, University of Kiel, Otto-Hahn-Platz 9, 24118 Kiel, Germany; 2 Institute of Pathology, Otto-von-Guericke University Magdeburg, Leipziger Str. 44, 39120 Magdeburg, Germany; 3 Institute for Medical Microbiology, Virology and Hygiene, University Medical Center Eppendorf, 20246 Hamburg, Germany

**Keywords:** interleukin, IL-6, IL-11, trans-signaling, metalloproteases, ADAM, MMP, meprin

## Abstract

Interleukin-11 (IL-11) has been associated with inflammatory conditions, bone homeostasis, hematopoiesis, and fertility. So far, these functions have been linked to classical IL-11 signaling via the membrane bound receptor (IL-11R). However, a signaling cascade via the soluble IL-11R (sIL-11R), generated by proteolytic cleavage, can also be induced. This process is called IL-11 trans-signaling. A disintegrin and metalloprotease 10 (ADAM10) and neutrophil elastase were described as ectodomain sheddases of the IL-11R, thereby inducing trans-signaling. Furthermore, previous studies employing approaches for the stimulation and inhibition of endogenous ADAM-proteases indicated that ADAM10, but not ADAM17, can cleave the IL-11R. Herein, we show that several metalloproteases, namely ADAM9, ADAM10, ADAM17, meprin β, and membrane-type 1 matrix metalloprotease/matrix metalloprotease-14 (MT1-MMP/MMP-14) when overexpressed are able to shed the IL-11R. All sIL-11R ectodomains were biologically active and capable of inducing signal transducer and activator of transcription 3 (STAT3) phosphorylation in target cells. The difference observed for ADAM10/17 specificity compared to previous studies can be explained by the different approaches used, such as stimulation of protease activity or making use of cells with genetically deleted enzymes.

## 1. Introduction

IL-11 is a crucial factor in several physiological and pathophysiological signaling pathways [1]. It plays an important role in inflammation, osteogenesis, hematopoiesis, and fertility [1,2,3]. So far, those functions have only been linked to interleukin-11 (IL-11) signaling via the membrane bound IL-11 receptor (IL-11R). In this so-called classic signaling pathway, IL-11 binds to its membrane bound α-receptor, inducing the dimerization of the ubiquitously expressed β-receptor glycoprotein-130 (gp130) on the same cell. This complex then activates intracellular JAK/STAT, MAPK, PI3K signaling pathways [4,5,6]. The IL-11R can be released from the cell surface [7] by proteolytic ectodomain-shedding, which is analogous to IL-6R solubilization [8]. Soluble receptors such as sIL-6R and sIL-11R can then bind their ligands, IL-6 or IL-11, respectively, before inducing dimerization of gp130 on distinct cells. This pathway is called trans-signaling. sIL-11R can be found in serum of healthy individuals [7], strongly suggesting a physiological function for IL-11 trans-signaling, as known for sIL-6R [9].

Similar to the IL-6R [10,11], the IL-11R consists of three extracellular domains, an immunoglobulin (Ig)-like domain (D1), two fibronectin type III domains (D2 and D3), a stalk region, which is a 54 amino acid long stretch connecting the extracellular part to the transmembrane region (TM), followed by an intracellular domain (ICD) [11,12]. The extracellular domains D2 and D3 form the cytokine binding module, which is essential for signaling [9].

Ectodomain-shedding of the IL-11R and IL-6R requires proteolytic cleavage of the receptors within their stalk regions. IL-6 trans-signaling can be proteolytically induced by a disintegrin and metalloprotease 10 (ADAM10) and ADAM17, as well as by meprin α and meprin β [13]. IL-11R shedding has been attributed to ADAM10 and the neutrophil-derived serine protease neutrophil elastase (NE), thereby inducing trans-signaling [7].

Cleavage of the IL-6R by its major sheddase ADAM17 takes place between P^355^ and V^356^ [14,15]. For meprin β-mediated cleavage of the IL-6R, the cleavage site at P^355^/V^356^ is in too close proximity to the cell surface and thus sterically not accessible [13]. Additionally, the meprin β-generated sIL-6R fragment has a molecular weight of about 50 kDa, which is different from the 70 kDa fragment produced by ADAM17. This indicates that the meprin β cleavage site in the IL-6R stalk region is further N-terminal, as compared to the one used by ADAM17 [13]. So far, no precise cleavage site for the IL-11R has been identified. However, experiments with deletion variants of the IL-11R indicated that R^355^ in the IL-11R stalk region is important for ADAM10-mediated shedding [7].

ADAM10 and ADAM17 are ubiquitously expressed membrane bound metalloproteases promoting important biological functions. ADAM10 is an essential factor in Notch signaling and ADAM10 knock-out mice resemble the phenotype of Notch deficient animals [16]. ADAM17 plays an essential role in inflammation and cell proliferation by releasing TNFα and ligands of the EGFR [17]. Structurally, ADAM10 and ADAM17 are closely related and exhibit a characteristic domain composition comprising a N-terminal signal peptide followed by a prodomain, a metalloprotease domain, a disintegrin domain, a cysteine-rich domain, a single transmembrane helix, and a cytoplasmic region [18]. ADAM9 is another member of the ADAM family of proteases. It has the same domain topology, except for an EGF-like domain between the cysteine-rich domain and the transmembrane helix [17].

The metalloproteases meprin α and meprin β are multidomain oligomeric metalloproteases playing critical roles in inflammation and maturation of fibrillar collagen [19,20,21]. Meprin α is cleaved by furin on the secretory pathway and is thus released as a soluble protease, whereas pro-meprin β is expressed as a membrane bound protein and can be activated by matriptase-2 or the bacterial protease RgpB at the cell surface [22,23,24]. In its inactive proform, meprin β can also be shed from the cell surface by ADAM10 and ADAM17 and subsequently becomes activated by tryptic proteases [24,25,26,27]. Prominent substrates of meprins are CD99 [28,29], procollagen [20], mucine 2 [30], as well as IL-6 [31], and the IL-6R [13], highlighting their role in inflammation and extracellular matrix homeostasis and indicate a partially shared substrate pool with ADAM proteases.

Membrane-type 1 matrix metalloprotease (MT1-MMP) as a member of the MMP-family seems to act as a counterpart to meprins by degrading fibrillary collagens I, II, III. It also cleaves ADAM9 [32].

Meprins, as astacin-proteases, together with ADAMs and MMPs, belong to the metzincin superfamily. As described above, these proteases have a partially overlapping substrate pool [19,33,34,35]. The IL-6R and IL-11R are cleaved within their respective stalk regions [7,13]. The length and amino acid composition of these regions indicate broad susceptibility for proteolysis of both receptors. Hence, we hypothesized that IL-6R and IL-11R are susceptible for shedding by a larger group of proteases.

In this study we employed genetically-modified HEK293T cells deficient for ADAM10 and ADAM17 to avoid basal shedding of the IL-11R by these well-described sheddases. In this proof-of-concept study we were able to specifically address the capability of certain metalloproteases to cleave the IL-11R and the IL-6R. This would not have been possible in the HEK293T wild-type cell-line, which was often used to study ADAM10/17 activity after stimulation with ionomycin or phorbol myristate acetate (PMA), respectively. These compounds however are not solely influencing ADAM10/17 and we therefore compared in a proof-of-concept approach stimulation of endogenous ADAM proteases in HEK293T wild-type cells with retransfection of ADAM10/17 and other metalloproteases on an ADAM10/17-free background.

## 2. Results

### 2.1. The IL-11R Is Cleaved by Different Metalloproteases

**Figure 1:**
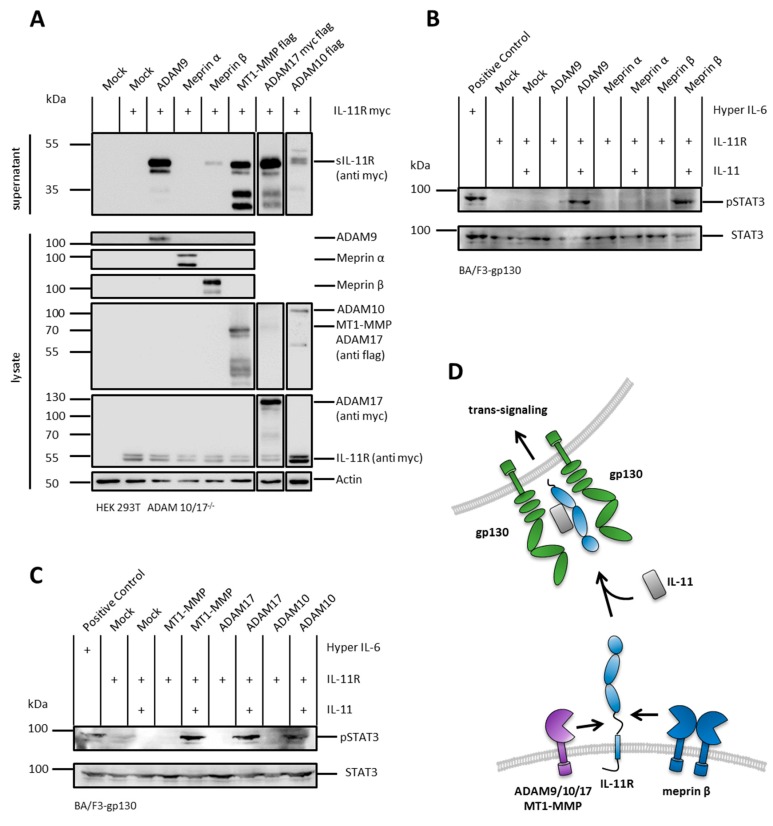
The membrane bound interleukin-11 receptor (IL-11R) is shed by different metalloproteases and induces interleukin-11 (IL-11) trans-signaling. (**A**) Shedding of the IL-11R in HEK293T ADAM 10/17^−/−^ cells. Cells were transfected with a N-terminally myc-tagged variant of the IL-11R alone or with ADAM9, meprin α, meprin β, membrane-type 1 matrix metalloprotease (MT1-MMP), ADAM17, or ADAM10. Supernatants were ultracentrifuged, trichloroacetic acid (TCA)-precipitated, and analyzed by immunoblotting with an antibody raised against the myc-tag. ADAM9, meprin α, and meprin β were detected with specific antibodies in lysate controls. MT1-MMP was flag-tagged, IL-11R was myc-tagged, and ADAM17 flag-/myc-tagged. pcDNA3.1 served as mock and actin as loading control. Cotransfection of IL-11R and ADAM10 was performed in an independent experiment. (**B**) Phosphorylation of STAT3 in Ba/F3-gp130 cells stably transfected with gp130. Cells were treated with supernatants from the experiments in (**A**) and with recombinant IL-11. Phosphorylation of STAT3 was analyzed for cotransfection experiments of IL-11R with ADAM9, meprin α, and meprin β. A fusion protein consisting of soluble IL-6R and IL-6 (Hyper IL-6) served as positive control for pSTAT3. Mock control from (**A**) served as negative control. Phosphorylation was detected with an antibody raised against phosphorylated STAT3. Total STAT3 protein served as loading control. (**C**) Same experimental setting as in (**B**) but for MT1-MMP, ADAM17, and ADAM10 as IL-11R sheddases. (**D**) Schematic overview of IL-11R shedding by ADAM9, ADAM10, ADAM17, MT1-MMP, and meprin β.

The IL-11R can, at least in vitro, signal through its membrane bound as well as its soluble form. ADAM10 and neutrophil elastase have been described as sheddases of the IL-11R [7]. We hypothesized that, due to the length of the stalk region and its amino acid composition, the IL-11R should potentially be prone to proteolysis by additional proteases. To further investigate the production of sIL-11R by proteolysis, we transiently transfected human embryonic kidney 293T cells deficient for ADAM10 and ADAM17 (HEK293T ADAM 10/17^−/−^) with cDNAs coding for IL-11R and different metalloproteases. Indeed, upon cotransfection with ADAM9, meprin β, MT1-MMP, ADAM17, and ADAM10, we could detect sIL-11R in the cell culture supernatant, demonstrating that all these proteases are putative sheddases of the IL-11R (Figure 1A). Of note, among the tested proteases only cotransfected meprin α showed no activity towards the IL-11R.

### 2.2. sIL-11R Shed by Different Metalloproteases Is Able to Induce Phosphorylation of STAT3

It has recently been shown that a complex of IL-11 and sIL-11R is able to induce the JAK/STAT-pathway via phosphorylation of STAT3 [7]. We tested sIL-11R generated by different proteases for biological activity on Ba/F3-gp130 cells. Therefore, we treated these cells with the conditioned media resulting from the cotransfection experiments and with recombinant IL-11. In a second step, the cell lysates were stained for phosphorylated STAT3 to validate biological activity of the IL-11R ectodomains.

Indeed, sIL-11R resulting from cotransfection experiments with the proteases ADAM9, meprin β, MT1-MMP, ADAM17, or ADAM10 (Figure 1A) induced phosphorylation of STAT3, thus proving the ability to induce IL-11 trans-signaling (Figure 1B–D).

### 2.3. ADAM 10 and ADAM 17 Are Both Able to Shed the IL-11R

**Figure 2:**
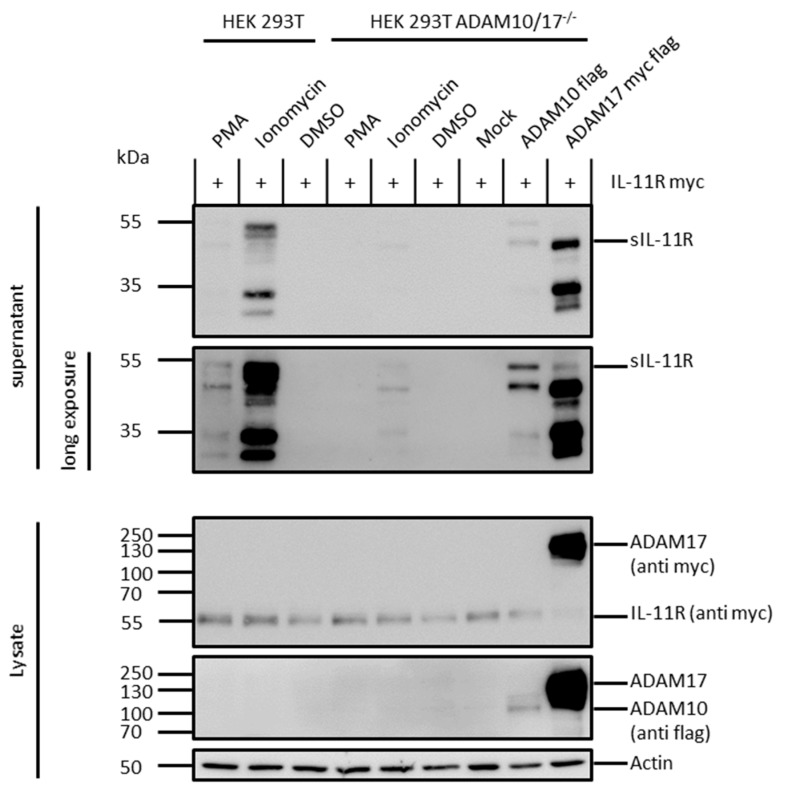
ADAM10 retransfected in HEK293T ADAM 10/17^−/−^ cells is capable of shedding the IL-11R. HEK293T wild-type cells (lanes 1–3) and HEK293T ADAM10/17^−/−^ cells (lanes 4–6) were transfected with a N-terminally myc-tagged variant of the IL-11R and stimulated with phorbol myristate acetate (PMA) or ionomycin. Dimethyl sulfoxide (DMSO) served as control. HEK293T ADAM10/17^−/−^ were transfected with an N-terminally myc-tagged variant of the IL-11R and ADAM10 or ADAM17 (lanes 7–9). For both setups the supernatant was ultracentrifuged, TCA-precipitated, and analyzed by immunoblotting with different antibodies. ADAM10 flag-tagged, IL-11R myc-tagged, and ADAM17 flag-/myc-tagged were detected in lysate controls. pcDNA3.1 served as mock and actin as loading control.

To examine cleavage of the IL-11R by ADAM proteases, we either stimulated endogenous ADAM proteases in HEK293T wild-type cells transiently transfected with the IL-11R, or we retransfected HEK293T ADAM 10/17^−/−^ cells with ADAM10 or ADAM17 together with IL-11R. Upon stimulation with the phorbolester PMA, a strong inducer of ADAM17 activity via activation of Protein Kinase C, shedding of the IL-11R was slightly increased (Figure 2) [36]. Stimulation with ionomycin, which activates ADAM10 via cellular influx of calcium, also induced shedding, albeit to a much higher extent. This implies that ADAM10 is the major sheddase of the IL-11R in HEK293T wild-type cells, as described previously [7]. ADAM17 on the other hand sheds the IL-11R only to a much lower extent. However, upon retransfection of HEK293T ADAM 10/17^−/−^ cells with cDNAs coding for the proteases ADAM10 and ADAM17, shedding of the IL-11R was observed (Figure 2). Interestingly, ionomycin also induced IL-11R shedding in the HEK293T ADAM 10/17^−/−^ cells without retransfection of proteases, indicating that not only ADAM10/17, but also other sheddases of the IL-11R exist, at least in the cell line used herein.

### 2.4. Arginine 355 Is not Obligatory for Ectodomain Shedding of the IL-11R

**Figure 3:**
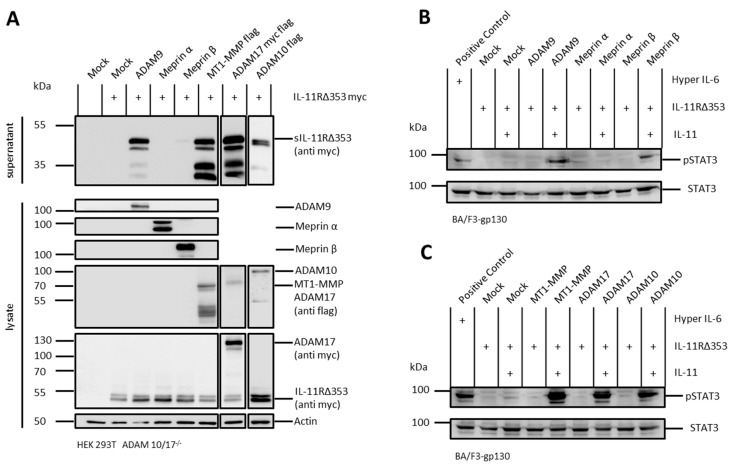
Shedding of the IL-11RΔ353 in HEK293T ADAM10/17^−/−^ cells. (**A**) HEK293T ADAM10/17^−/−^ cells were transfected with a N-terminally myc-tagged variant of the IL-11RΔ353 and ADAM9, meprin α, meprin β, MT1-MMP, ADAM17, or ADAM10. Supernatants were ultracentrifuged, TCA-precipitated, and analyzed by immunoblotting with different antibodies. pcDNA3.1 served as mock and actin as loading control. Cotransfection of IL-11RΔ353 and ADAM10 was performed in an independent experiment. (**B**) Phosphorylation of STAT3 in Ba/F3-gp130 cells stably transfected with gp130. Cells were treated with supernatants from the experiments in (**A**) and with recombinant IL-11. Phosphorylation of STAT3 was analyzed for cotransfection of IL-11RΔ353 with ADAM9, meprin α, and meprin β. A fusion protein consisting of soluble IL-6R and IL-6 (Hyper IL-6) served as positive control. Mock control from (**A**) served as negative control. Phosphorylation was detected with an antibody raised against phosphorylated STAT3. Total STAT3 protein served as loading control. (**C**) Same experimental setting as in (**B**) but for MT1-MMP, ADAM17, and ADAM10 as IL-11RΔ353 sheddases.

The cotransfection experiments described above were repeated with an IL-11R variant lacking amino acids H353-S362 (IL-11RΔ353). The amino acid Arg355 is located within the stalk region, which was previously described to be critical for ADAM10-mediated shedding of the IL-11R, as a mutation of arginine to glutamic acid at this position resulted in significantly reduced proteolysis [7]. However, cotransfection of HEK293T ADAM 10/17^−/−^ cells with ADAM9, meprin β, MT1-MMP, ADAM17, or ADAM10 with IL-11RΔ353 resulted in shedding of this IL-11R variant (Figure 3A). Moreover, sIL-11RΔ353 was able to induce trans-signaling, as measured by pSTAT3 in stimulated Ba/F3-gp130 cells (Figure 3B,C).

### 2.5. The IL-6R Is Cleaved by Several Metalloproteases

**Figure 4:**
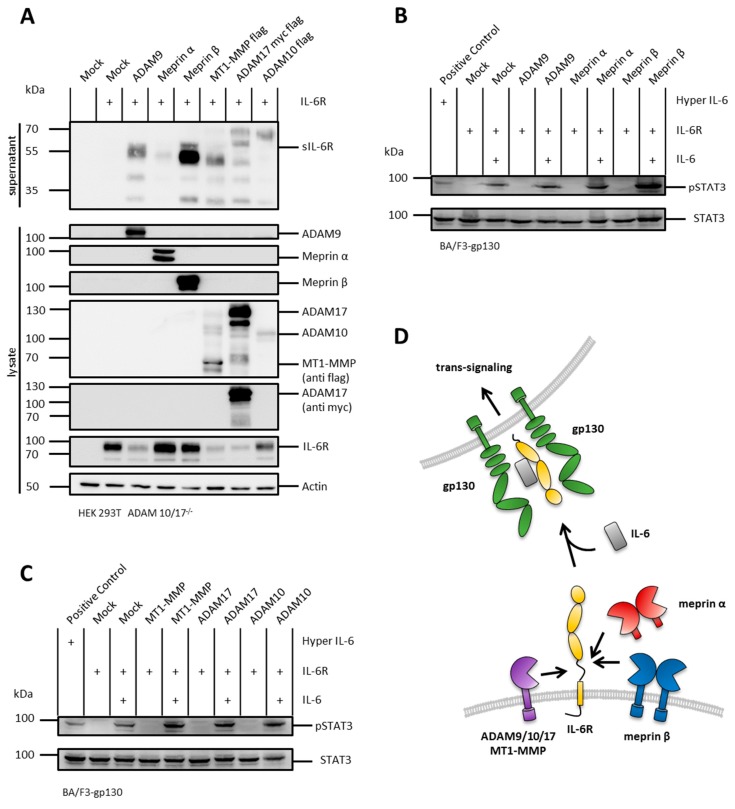
The IL-6R is shed by different metalloproteases and induces IL-6 trans-signaling. (**A**) HEK293T ADAM10/17^−/−^ cells were transfected with IL-6R and/or ADAM9, meprin α, meprin β, MT1-MMP, ADAM17, or ADAM10. Supernatants were ultracentrifuged, TCA-precipitated, and analyzed by immunoblotting with an antibody raised against the D1-domain of the IL-6R. ADAM9, meprin α, and meprin β were detected with specific antibodies in lysate controls. MT1-MMP and ADAM10 are flag-tagged and ADAM17 flag-/myc-tagged and detected respectively in lysate controls. pcDNA3.1 served as mock and actin as loading control. (**B**) Phosphorylation of STAT3 in Ba/F3-gp130 cells stably transfected with gp130. Cells were treated with supernatants from the experiments in (**A**) and with recombinant IL-6. Phosphorylation of STAT3 was analyzed for cotransfection with ADAM9, meprin α, and meprin β. A fusion protein consisting of soluble IL-6R and IL-6 (Hyper IL-6) served as positive control. Mock control from (**A**) served as negative control. Phosphorylation was detected with an antibody raised against phosphorylated STAT3. Total STAT3 protein served as loading control. (**C**) Same experimental setting as in (**B**) but for MT1-MMP, ADAM17, and ADAM10 as IL-6R sheddases. (**D**) Schematic overview of IL-6R shedding by ADAM9, ADAM10, ADAM17, MT1-MMP, meprin α, and meprin β.

It has been shown that, similarly to the IL-11R, the IL-6R can signal through its membrane bound as well as its soluble form. The sIL-6R can be, comparable to sIL-11R, the result of a proteolytic cleavage event induced by ADAMs or meprins [7,13]. To analyze the production of sIL-6R by proteolysis, we applied the same experimental setting as for the cotransfection with the IL-11R. Upon cotransfection with ADAM9, meprin α, meprin β, MT1-MMP, ADAM17, or ADAM10, we could detect sIL-6R in the cell culture supernatants, thus demonstrating that the IL-6R can be shed by all these proteases (Figure 4A).

Similar to the above described IL-11R shedding and trans-signaling experiments (Figure 1A–D, Figure 3A–C), the proteases ADAM9, meprin α, meprin β, MT1-MMP, ADAM17, and ADAM10 induced phosphorylation of STAT3 by shedding of the IL-6R, thus proving the ability to also induce IL-6 trans-signaling (Figure 4A–D).

### 2.6. N-Terminal Cleavage of the IL-6R and the IL-11R Apart from the Stalk Regions

The results from the cotransfection experiments showed that at least two different shedding sites within the stalk region of the IL-6R can be assessed, which are in accordance with previous studies [13]. Conclusively, this resulted in a 70 kDa fragment produced by ADAM10 and ADAM17 and a 50 kDa fragment produced by ADAM9, meprin α, meprin β, MT1-MMP, and ADAM17 (Figure 4A). Of note, cotransfection with ADAM9, meprin β, MT1-MMP, ADAM10, or ADAM17 led to additional fragments of about 30 kDa and/or 40 kDa. Immunodetection of the cell culture supernatants was performed with an antibody raised against the D1-domain of the IL-6R. Therefore, these two fragments could be the product of cleavage events within the linker regions between domains D3 and D2, as well as D2 and D1.

Comparable to IL-6R-, also IL-11R cleavage results in multiple fragments. Upon stimulation of HEK293T wild-type cells, as well as cotransfection of HEK293T ADAM 10/17^−/−^ cells with different proteases, a characteristic pattern of at least four different receptor bands appeared (Figure 1A, Figure 2 and Figure 3A). This implied that there could be more than one cleavage site within the IL-11R. The two bands between 35 and 55 kDa likely represent shedding within the stalk region resulting in the full-length soluble receptor. The bands at 30 and 35 kDa could be due to cleavage in the linker region between extracellular domains D1 to D3. Analogous to the IL-6R, this band pattern underlines cleavage specificities for the different proteases.

## 3. Discussion

The pro-inflammatory cytokines IL-6 and IL-11 fulfill several important functions. IL-6 is an important mediator in the innate and acquired immune system, involved in differentiation of T-cells, release of c-reactive protein [9], and acts as acute phase trigger [6]. IL-6 trans-signaling is driven by ectodomain shedding of the IL-6R and has been shown to be a critical event in inflammatory diseases such as rheumatoid arthritis and inflammatory bowel disease [9]. IL-11 is involved in hematopoiesis, especially production of thrombocytes, which led to its approval as the drug ‘Oprelvekin’ for the treatment of chemotherapy-induced thrombocytopenia [7]. Additionally, IL-11 can activate the hepatic acute phase response [37]. IL-11R knock-out mice underlined an important role in fertility [2] and bone homeostasis [3], as IL-11R knock-out mice show elevated bone density. Mutations within the ectodomain of the IL-11R are associated with craniosynostosis [2,3] and gastric tumorigenesis [38,39]. Moreover, IL-11 is produced by osteoblasts, which further implies a possible role of IL-11 signaling in osteoporosis [40]. For IL-11 trans-signaling, no definite physiological or pathophysiological event has been defined so far, although, sIL-11R can be detected in the plasma of healthy individuals [7]. We could detect both IL-11R shedding and IL-11 trans-signaling in vitro, which strongly suggests an in vivo function for sIL-11R, as demonstrated for sIL-6R.

In this study, we demonstrated that ectodomain-shedding and subsequent induction of trans-signaling of the IL-6R and the IL-11R can be mediated by different metalloproteases. We hypothesize that the length and amino acid composition of both receptors are the reason for their broad susceptibility for proteolysis, which in turn underlines the importance of this trans-signaling event.

So far, possible sheddases of the IL-11R have been identified on the basis of stimulation experiments to induce proteolytic activity of endogenous ADAM10 and ADAM17 in HEK293T wild-type cells [7]. However, stimulation with PMA or ionomycin may additionally induce other proteases besides ADAM10/17 potentially cleaving the IL-11R and IL-6R. To address this issue in a genetically modified system, we employed HEK293T cells deficient for ADAM10 and ADAM17 and retransfected these cells with the proteases and the IL-11R, which allowed us to associate the shedding events precisely with the transfected proteases. Of note, retransfection of the genetically-modified HEK293T cells resulted in some experiments in slightly higher expression levels of ADAM10/17 compared to HEK293T wild-type cells. However, we used this system in a proof-of-concept approach to demonstrate the biochemical capability of certain proteases to cleave the IL-11R and IL-6R. Indeed, we observed shedding of the IL-6R and the IL-11R by several metalloproteases, which resulted in receptor fragments of different sizes, indicating cleavage events in different regions within the receptors. For the IL-6R, two different shedding sites within the stalk region have already been discussed [13]. However, two additional fragments appeared upon cotransfection of the IL-6R with ADAM9, meprin β, MT1-MMP, or ADAM17. We hypothesize that those fragments represent cleavage in the linker regions in between the ectodomains D3 and D2, as well as D2 and D1. Meprin α seemed to solely target the stalk region of the IL-6R, resulting in a fragment of about 50 kDa, whereas ADAM10 seemed to cleave within the stalk region as well as within the linker region between domains D2 and D1.

A comparable band pattern occurred in the cotransfection with the IL-11R. The bands at 30 and 35 kDa could represent cleavage events in the linker region between extracellular domains D1 to D3. Analogous to the IL-6R, this band pattern underlines different cleavage specificities for the tested proteases.

Overall, we observed clear differences between the pharmacological and genetically-modified approaches, which has to be considered, particularly with regard to therapeutic manipulation of IL-6/11 signaling.

In sum, the physiological and pathophysiological functions of IL-6 trans-signaling have been studied to a great extent in health and disease [1,9]. In this proof-of-concept study we showed that the IL-11 trans-signaling can be induced by ADAM9, meprin β, MT1-MMP, ADAM10, or ADAM17 in vitro. Future experiments will show if this concept also holds true in vivo. A possibility to address potential pathological impact of some of the proteases analyzed in this study would be the generation of knock-in mouse models to induce protease-specific expression in certain tissues under diseased conditions.

## 4. Material and Methods

### 4.1. Chemicals

All chemicals were of analytical grade and obtained from Carl Roth GmbH & Co. KG (Karlsruhe, Germany), Merck KGaA (Darmstadt, Germany), Sigma Aldrich Inc. (Darmstadt, Germany) and Thermo Fisher Scientific Inc. (Waltham, Massachusetts, USA), if not stated otherwise.

### 4.2. Cells

HEK293T wild-type (DSMZ GmbH, Braunschweig, Germany) and HEK293T ADAM 10/17^−/−^ cells (kindly provided by Dr. Björn Rabe [41] ) were maintained in Dulbecco’s modified eagle medium (DMEM; Gibco^TM^, Waltham, Massachusetts, USA) with GlutaMAX^TM^ supplemented with 10% (*v*/*v*) FCS, 100 units/L penicillin, 100 µg/mL streptomycin, and 50 µg/mL Gentamycin.

Ba/F3-gp130 cells [42], which have been stably transfected with gp130 cDNA and therefore respond to the IL-6/sIL-6R or IL-11/sIL-11R complexes [43], were maintained in Dulbecco’s modified eagle medium (DMEM; Gibco^TM^) with GlutaMAX^TM^ supplemented with 10% (*v*/*v*) FCS, 100 units/L penicillin, 100 µg/mL streptomycin, 50 µg/mL Gentamycin, and 10 ng/mL Hyper-IL-6. All cells were kept under humidified conditions (5% CO_2_) at 37 °C.

### 4.3. Plasmids

- pcDNA 3.1
- murine ADAM9 in pcDNA 3.1
- human ADAM10 flag-tagged in pcDNA4/TO
- human ADAM17 DDK in pCMV6: purchased on Origene
- human meprin α in pSG5
- human meprin β in pSG5
- human IL-11R N:myc, C:HA in pcDNA3.1
- human IL-11R in pcDNA 3.1 lacking H353-S362
- human MT1-MMP flag-tagged in pcDNA4/TO
- human IL-6R in pcDNA 3.1
- Sequences were confirmed by Sanger-sequencing.

### 4.4. Shedding Assay

A total of 2.5–3 × 10^6^ cells per dish were transiently transfected with an N-terminally myc and C-terminally HA-tagged variant of the IL-11R or different proteases and the IL-11R. pcDNA 3.1 alone was used as mock-control. In the same experimental setting, a N-terminally myc-tagged IL-11R variant lacking amino acids H353-S362 was cotransfected with the proteases. Polyethylenimine (PEI, Polysciences Europe GmbH) was used as transfection-reagent. For cell transfections, plasmids and PEI were mixed in a ratio of 1:3 in serum-free medium. After 30 min of incubation at room temperature, the transfection-mixture was added to the cell culture in a total volume of 5 mL. After incubation for 5 h, an additional 5 mL of medium was added and incubation was continued overnight. Cell culture medium was replaced with serum-free medium for additional 24 h. For the detection of soluble receptors, the supernatant was collected and cleared by centrifugation (500× *g*, 10 min, room temperature). Additionally, the cell culture supernatant was ultracentrifuged at 186,000× *g* for 2 h at 4 °C. Cell culture supernatants were normalized to the amount of protein of the respective cell lysates and analyzed by immunoblotting. For concentration of proteins, respective cell culture supernatants were precipitated with trichloroacetic acid (TCA 10% (*w*/*v*)) following incubation on ice for 60 min. Afterwards proteins were pelleted (15,000× *g*, 15 min, 4 °C), washed with ice cold acetone, and dissolved in sample-buffer.

### 4.5. ADAM10/17 Stimulation

HEK293T wild-type and HEK293T ADAM10/17^−/−^ cells were transfected (as described above) with the IL-11R. Afterwards, cells were washed twice with sterile PBS and cell culture medium was replaced with serum-free medium. Cells were then stimulated by adding PMA (100 nM) for 2 h or ionomycin (1 µM) for 1 h. Dimethyl sulfoxide (DMSO) served as control. Cells were harvested and lysed (as described below) and cell culture supernatants were analyzed (as described previously) [7].

### 4.6. Phosphorylation Assay

Ba/F3-gp130 cells were washed four times with sterile PBS and incubated in serum-free medium for 2 h. A total of 10^6^ cells per vial were suspended in 300 µL conditioned medium (shedding assay) and either 150 ng recombinant IL-11 or 150 ng Hyper-IL-6 [7,13] was added. Cells were incubated at 37 °C, shaking at 500 rpm, for 15 min before centrifugation at 500× *g* for 3 min at room temperature. The supernatant was discarded, and the cell-pellet was boiled in sample-buffer. SDS-PAGE and immunoblot analysis were performed to analyze protein expression and protein phosphorylation.

### 4.7. Cell Lysis, SDS-PAGE, and Immunoblot Analysis

Transfected cells were harvested in ice-cold PBS and centrifuged at 1000× *g* for 10 min at 4 °C. Cell pellets were washed three times with PBS prior to resuspension in lysis buffer (cOmplete ^TM^ protease inhibitor cocktail, 1% (*v*/*v*) Triton-X 100, PBS, pH 7.4) before incubation on ice for 45 min. Afterwards, the cell-suspension was centrifuged at 15,000× *g* at 4 °C for 15 min and the protein amount was determined using a BCA protein assay kit (Thermo Fisher Scientific Inc.). Cell culture supernatants were normalized to the protein content of the respective cell lysates to ensure a comparable analysis. Protein samples were boiled in sample buffer containing DTT. After separation by SDS-PAGE proteins were transferred on nitrocellulose membranes by blotting, which were then saturated with 5% dry milk or 3% BSA for 1 h at room temperature. The membranes were then incubated with primary antibody at 4 °C overnight. After washing in TBS, membranes were incubated with horseradish peroxidase-conjugated secondary antibodies (Thermo Fisher Scientific Inc.) in 5% dry milk or TBS for 1 h at room temperature. Chemiluminescence was detected using the Super Signal^TM^ West Femto kit (Thermo Fisher Scientific Inc.) with the LAS-3000 Imaging System (FUJIFILM Europe GmbH). The following antibodies were used for detection: anti-meprin α/meprin β (polyclonal Abs, generated against ectodomains; Pineda); anti-Myc (# 2276, Cell Signaling Technology, Inc., Cambridge, UK); anti-Flag (F1804, clone M2, Sigma-Aldrich); anti-ADAM9 (polyclonal Ab, kindly provided by Carl Blobel); anti-Actin (#097M4883V, Sigma-Aldrich Inc.); anti-IL-6R (4-11 monoclonal, generated against the D1-domain); and anti-pSTAT3 (#9131 (Tyr705), Cell signaling Technology, Inc.); anti-STAT3 (#9139 (124H6), Cell signaling Technology, Inc.).
